# Impact of MRI radiomic feature normalization for prognostic modelling in uterine endometrial and cervical cancers

**DOI:** 10.1038/s41598-024-66659-w

**Published:** 2024-07-22

**Authors:** Erlend Hodneland, Erling Andersen, Kari S. Wagner-Larsen, Julie A. Dybvik, Njål Lura, Kristine E. Fasmer, Mari K. Halle, Camilla Krakstad, Ingfrid Haldorsen

**Affiliations:** 1 MMIV Mohn Medical Imaging and Visualization Centre, Department of Radiology, Haukeland University Hospital, Jonas Lies Vei 65, 5021 Bergen, Norway; 2 Department of Mathematics, University of Bergen, Bergen, Norway; 3 Department of Clinical Engineering, Haukeland University Hospital, Bergen, Norway; 4 Department of Obstetrics and Gynecology, Haukeland University Hospital, Bergen, Norway; 5 Department of Clinical Science, Centre for Cancer Biomarkers, University of Bergen, Bergen, Norway; 6 Section for Radiology, Department of Clinical Medicine, University of Bergen, Bergen, Norway

**Keywords:** Radiomics, MRI, Endometrial cancer, Cervical cancer, Normalization, Cancer imaging, Gynaecological cancer

## Abstract

Widespread clinical use of MRI radiomic tumor profiling for prognostication and treatment planning in cancers faces major obstacles due to limitations in standardization of radiomic features. The purpose of the current work was to assess the impact of different MRI scanning- and normalization protocols for the statistical analyses of tumor radiomic data in two patient cohorts with uterine endometrial-(EC) (n = 136) and cervical (CC) (n = 132) cancer. 1.5 T and 3 T, T1-weighted MRI 2 min post-contrast injection, T2-weighted turbo spin echo imaging, and diffusion-weighted imaging were acquired. Radiomic features were extracted from within manually segmented tumors in 3D and normalized either using z-score normalization or a linear regression model (LRM) accounting for linear dependencies with MRI acquisition parameters. Patients were clustered into two groups based on radiomic profile. Impact of MRI scanning parameters on cluster composition and prognostication were analyzed using Kruskal–Wallis tests, Kaplan–Meier plots, log-rank test, random survival forests and LASSO Cox regression with time-dependent area under curve (tdAUC) (α = 0.05). A large proportion of the radiomic features was statistically associated with MRI scanning protocol in both cohorts (EC: 162/385 [42%]; CC: 180/292 [62%]). A substantial number of EC (49/136 [36%]) and CC (50/132 [38%]) patients changed cluster when clustering was performed after z-score-versus LRM normalization. Prognostic modeling based on cluster groups yielded similar outputs for the two normalization methods in the EC/CC cohorts (log-rank test; z-score: p = 0.02/0.33; LRM: p = 0.01/0.45). Mean tdAUC for prognostic modeling of disease-specific survival (DSS) by the radiomic features in EC/CC was similar for the two normalization methods (random survival forests; z-score: mean tdAUC = 0.77/0.78; LRM: mean tdAUC = 0.80/0.75; LASSO Cox; z-score: mean tdAUC = 0.64/0.76; LRM: mean tdAUC = 0.76/0.75). Severe biases in tumor radiomics data due to MRI scanning parameters exist. Z-score normalization does not eliminate these biases, whereas LRM normalization effectively does. Still, radiomic cluster groups after z-score- and LRM normalization were similarly associated with DSS in EC and CC patients.

## Introduction

By extracting quantitative features from medical images, radiomic tumor profiling provides non-invasive biomarkers that have been shown to predict clinical phenotype relevant for clinical decision-making in several cancers, including endometrial-^1–3^, cervical-^4,5^, breast-^6,7^, lung-^8^, and brain^9^ cancers. Tumor radiomic features have also been linked to molecular tumor markers representing putative targets for novel treatments^2^. Despite showing promising results in retrospective studies for guiding personalized diagnosis, prognostication, and treatment, radiomics has yet to be widely adopted in routine clinical practice. Until recently, lack of robust and automated methods for tumor segmentation has impeded clinical integration of radiomics. However, promising deep learning-based automated tumor segmentation methods are being developed, e.g. in endometrial and cervical cancer^10,11^. Another hurdle to clinical implementation of radiomics is the limitations in reproducibility of radiomic features^12–15^ and lack of robust infrastructure for integrating radiomics with other multimodal imaging-genomic- and clinical data currently guiding choice of treatment.

To ensure effectiveness of radiomic models and their potential clinical utility, it is crucial to manage biases. Otherwise, there is a risk of overfitting and encountering false discoveries, which can limit the models' generalizability and validity. For MRI radiomics, a main challenge is that field strength and vendor- and sequence variations will affect the MRI signal, and subsequently also the extracted radiomic feature values. MRI image contrast and signal intensities are inherently affected by some of the MRI protocol parameters. MRI signal correlates with magnetic field strength, and signal-to-noise ratio (SNR) with magnetic field strength and voxel size^16^. Differences in MRI scanners and protocols may thus lead to variations in extracted radiomic feature values.

A few previous phantom studies have investigated how variations in scanning protocol impact MRI radiomic feature values^17,18^. Lee et al.^17^ used T1- and T2-weighted imaging with different MRI scanner settings, i.e. slice thickness, number of excitations, field of view, and found that several of the extracted radiomic features had low reproducibility and high variability across MRI settings. Similarly, Yuan et al.^18^ found that variations in T2-weighed MRI parameters caused high variability in radiomic features, recommending radiomic features with low reproducibility to be used with caution and advocating harmonization of imaging protocols prior to potential implementation of radiomics in clinical use.

Concerns related to limitations in reproducibility of radiomic features have been discussed. The Image Biomarker Standardization Initiative (IBSI) represents a community-driven standard for radiomics, aiming to enhance the reproducibility of radiomic feature extraction as a step towards standardization^19^. However, even with standardized definitions, the distributions of radiomic features can still vary due to factors such as MRI scanning hardware and protocol settings, highlighting the complexity of harmonizing radiomic features. A widely used normalization method in MRI is z-score normalization^15,20, 21^, treating every radiomic feature value equally using global scaling factors. However, scanning variations will not be accounted for using global normalization methods. Other proposed methods are robust z-score, log-transformation, quantile, upper quartile, and whitening^22^. More advanced normalization methods, such as ComBat, developed for genomics to mitigate batch effects, have been proposed also for normalization of radiomics data^23^. However, concerns have been raised that ComBat may introduce new biases under certain conditions^24^.

The aim of this study was to compare MRI radiomic tumor profiles using two different normalization methods (z-score- and linear regression model (LRM) normalization) in two well-annotated uterine endometrial- and cervical cancer patient series. The objective was also to explore how the different normalization methods affect the radiomic clustering of patients. prognostication by cluster groups and the analyses of time-dependent disease-specific survival (DSS).

## Materials and methods

### Patient cohort and inclusion criteria

The local Institutional Review Board approved this retrospective study (#2015/2333/REK vest). All patients included in the study gave their written informed consent at the time of their primary diagnosis. We confirm that all procedures in the study are in accordance with relevant guidelines and regulations and the ethical standards of the 1964 Helsinki declaration and its later amendments. The patients were selected from a larger cohort of endometrial and cervical cancer patients who underwent pre-treatment pelvic MRI during 2009–2017. All included patients had histologically confirmed endometrial- or cervical cancer with visible primary tumor on pelvic MRI confirmed by two radiologists. See Table 1 for clinical and pathological characteristics of the patient cohorts.

**Table 1:** Clinical- and pathological characteristics in endometrial (primary MR between 2009 and 2019) and cervical (primary MR between 2009 and 2017) cancer patients.

	Endometrial cancer cohort (n = 136)	Cervical cancer cohort (n = 132)
Age at treatment, median (IQR)	68 (52, 84)	48 (26, 70)
Follow-up time (months), median (IQR)	60 (25, 94)	75 (32, 118)
Dead from endometrial/cervical cancer, n (%)		
Yes	21 (15)	33 (25)
Other	115 (85)	99 (75)
FIGO stage^a^, n (%)		
I	106 (79)	40 (30)
II	10 (7)	30 (23)
III	17 (13)	47 (36)
IV	2 (1)	15 (11)
Unknown	1 (1)	
Histologic grade EC, n (%)		
I	111 (85)	
II	24 (18)	
Unknown	1 (1)	
Histologic grade CC, n (%)		
I&II		102 (77)
III		22 (17)
Unknown		8 (6)
Histologic type EC, n (%)		
Endometroid	111 (82)	
Clear cell	6 (4)	
Serous papillary	10 (7)	
Carcinocarsoma	5 (4)	
Undifferentiated	3 (2)	
Unknown	1 (1)	
Histologic type CC, n (%)		
Squamous-cell carcinomas		103 (78)
Adenocarcinoma		21 (16)
Other		8 (6)

^a^Endometrial cancer patients: 2009 FIGO staging of carcinoma of the endometrium; Cervical cancer patients: 2018 FIGO staging of cancer of the cervix uteri. *CC* cervical cancer, *EC* endometrial cancer, *FIGO* International Federation of Gynecology and Obstetrics, *IQR* interquartile range.

### MRI acquisitions

Further inclusion criteria for the diagnostic pelvic MRI scans were the presence of (i) contrast-enhanced T1-weighted imaging (in endometrial cancer patients only), (ii) T2-weighted imaging (in all patients), and (iii) diffusion-weighted imaging (DWI) (in all patients). For contrast-enhanced T1-weighted imaging, gadolinium (Dotarem from Guerbet, France) was administered intravenously at a dosage of 0.1 mmol per kilogram of body weight and 2 min contrast delay, using an axial/axial oblique (relative to the long axis of the uterus) 3D volumetric interpolated breath-hold gradient echo sequence with fat saturation (VIBE + C). T2-weighed sequences and DWI were acquired in an axial/axial oblique plane. From the DWI sequence, apparent diffusion coefficient (ADC) maps were generated using the scanner software provided by the vendor. The highest b-value image from the DWI, referred to as "high-b", was also extracted (800 s·mm^−2^ ≤ high-b ≤ 4000 s·mm^−2^). MRI images were stored in the Digital Imaging and Communications in Medicine (DICOM) format (https://www.dicomstandard.org). Variations in scanner protocol settings were larger in the cervical cancer cohort than in the endometrial cancer cohort (Supplementary Table S1). For the endometrial cancer patients (n = 136), all had an MRI examination at the same hospital on Siemens scanners (1.5 T/3 T: n = 71/65). For the cervical cancer patients (n = 132), the MRI examinations were from three different hospitals on scanners from GE Medical Systems (1.5 T: n = 10), Philips Medical Systems (1.5 T/3 T: n = 31/9), and Siemens (1.5 T/3 T: n = 54/28).

MRI scanning parameters were extracted from the DICOM header of each MRI series. Scanning parameters of the DWI sequence were used for normalization of both ADC- and high-b derived radiomic features. The following primary MRI scanning parameters were extracted from the MRI sequences VIBE + C, T2, and DWI [physical unit is provided whenever given]: Spacing (h, equal in x- and y-direction and therefore not duplicated) [mm], matrix (m, equal in x and y-direction and therefore not duplicated), repetition time (TR) [ms], echo time (TE) [ms], flip angle (FA) [°], slice thickness (ST) [mm], interslice gap (IG) [mm], number of slices (NoS), field strength [T], number of averages (only for T2 and DWI), phase encoding direction (COLUMN or ROW), and b-values [s·mm^−2^] (only for DWI). The following secondary scanning variables were derived from the primary variables: Voxel volume = h^2^·ST [mm^3^], anisotropy = h/ST, field of view = (h·m)^2^·(ST + IG)·NoS [mm^3^], high-b [s·mm^−2^], and number of b-values (Supplementary Table S1).

### Manual tumor segmentation

**Figure 1:**
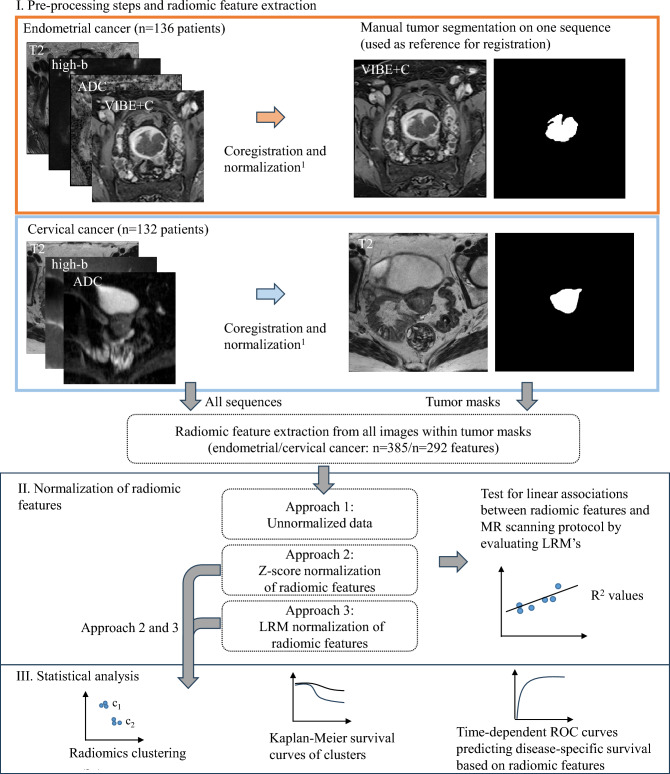
(**a**) The MRI sequences (VIBE + C, T2, ADC, and high b-value for endometrial cancer patients; T2, ADC and high b-value for cervical cancer patients) were co-registered to a common sequence used for manual tumor segmentation [VIBE + C for endometrial cancer patients (red box) and T2 for cervical cancer patients (blue box)]. White segmentations in the right panel represent the manually outlined tumor masks. After co-registration and normalization, radiomic features were extracted from all sequences within the tumor mask regions. (**b**) Radiomic features were tested for linear associations with MRI scanning protocol parameters using the linear regression model in (2), reporting R^2^- and p-values of this model, indicating the strength and significance of the relationship. Testing for linear associations was performed for unnormalized-, z-score normalized-(Approach 1) and LRM normalized (Approach 2) data. (**c**) For each of the normalizations (Approach 1 and 2), patients were clustered into two radiomic groups based on radiomic features, and statistical differences between these groups in relation to MRI scanning protocol parameters were tested. The ability of this clustering to identify patients exhibiting different phenotypes was assessed using Kaplan–Meier survival plots. Time-dependent area under the ROC curve (tdAUC) for predicting disease-specific survival based on radiomic features were also plotted. *ADC* apparent diffusion coefficient, *LRM* linear regression model, *ROC* receiver operating characteristic, *T2* T2 weighted MRI, *VIBE* + *C* T1 weighted MRI with contrast. ^1^z-score normalization.

Figure 1 summarizes the main steps of the algorithm for radiomic feature extraction. A total of 136 endometrial and 132 cervical cancer patients were used for 3D tumor segmentation. The endometrial cancer patients that underwent manual segmentation were randomly picked from all patients meeting the inclusion criteria (Fig. 1a, red box). All cervical cancer patients meeting the inclusion criteria were manually segmented (Fig. 1a, blue box). Three experienced radiologists manually outlined the boundaries of the primary tumor in 3D using ITKsnap v.3.6.0 (www.itksnap.org) (Endometrial cancer cohort: J.A.D: n = 77, K.W.L: n = 59; Cervical cancer cohort: N.L: n = 65, K.W.L: n = 67). For endometrial cancer patients, the segmentation was performed on axial/paraxial VIBE + C images, and for cervical cancer patients on axial/paraxial T2-weighted images. The other MRI sequences provided complementary information about tumor extent, aiding in the placement of tumor masks. In subgroups of the endometrial and cervical cancer cohorts, MRI tumor segmentations have previously been performed by two radiologists. This demonstrated moderate to high interrater agreement for whole-volume tumor segmentations with Dice scores for the two raters of 0.89 (in (34/136 patients)^10^ and 0.78 (in 26/132 patients)^11^ in endometrial- and cervical cancers, respectively.

### Radiomic feature extraction

MRI images in DICOM format were loaded and manipulated using the open-source software package Imagedata^25^. In the endometrial cancer cohort, T2 and DWI images (high-b and ADC maps) were spatially aligned with the VIBE + C image using the built-in “align” tool based on the geometric transform in the DICOM header. In the cervical cancer cohort, DWI images were aligned with the T2 images. After alignment, all images of the same patient were defined on the same grid with equal voxel size.

The images underwent z-score normalization prior to feature extraction, and then multiplied by a scaling factor of 100 and rounded to obtain images within a standard range of whole number values (integers). Whole tumor radiomic features were extracted from within the manually segmented tumor mask using the open source radiomics software Pyradiomics v3.0.1^26^ with default settings except from binWidth = 15, tuned to fit the scaling factor of 100. For each cohort, patient, and data set (VIBE + C [only in endometrial cancer], T2, high-b, ADC), we extracted a total of 95 grayscale-based radiomic features belonging to the following feature groups: GLCM (Gray Level Co-occurrence Matric, n = 24 features), GLRLM (Gray Level Run Length Matrix, n = 16), GLDM (Gray Level Dependence Matrix, n = 14), GLSZM (Gray Level Size Zone Matrix, n = 16), NGTDM (Neighboring Graytone Difference Matrix, n = 5), first order statistics (n = 18). Shape features (n = 13), derived from the tumor mask, were extracted only once for each patient. In total, 385/292 radiomic features were extracted in the endometrial/cervical cancer cohorts (Fig. 1a, lower box).

### Normalization methods

Radiomic data comes at different scales, and preprocessing by z-score normalization is commonly used to standardize the input range. Z-score normalization, also known as z-normalization, transforms a variable $f$

1 $${N}_{z}\left(f\right)= \frac{f-\mu (f)}{\sigma (f)}$$

with mean $\mu (f)$ and standard deviation $\sigma (f)$ into a transformed variable ${N}_{z}\left(f\right)$ with zero mean and standard deviation of one. As a baseline approach, all the radiomic features were z-score normalized (Approach 1; Fig. 1b). In the present study, we investigate the dependency of radiomic features on different normalization techniques. With this aim, we represent linear dependencies between MRI scanning parameters and each of the radiomic variables $f$ using a multilinear regression model (LRM), e.g. thus minimizing

$${min}_{{b}_{i} }{\left|f-\widetilde{f}\right|}^{2}$$

2 $$for \widetilde{f}={b}_{0}+{b}_{1}{x}_{1}+\cdots +{b}_{m}{x}_{m}$$

over a set of coefficients b_i_, i = 0, 1, …, m (fitlm.m in MATLAB^27^)The predictor variables *x*_*i*_ represent the following primary or secondary derived MRI scanning variables over all patients in the endometrial or cervical cancer cohort: voxel volume, voxel anisotropy, TR, TE, FA, field of view, number of averages (equal for all VIBE + C series, and hence not used to fit a linear regression model for VIBE + C derived radiomic features), slice thickness, field strength, phase encoding direction, highest b-value, and number of b-values. Highest b-value and number of b-values are applicable to DWI derived radiomic features only, i.e. ADC and high b-value series. The MRI scanning parameters applied in model (2) are also listed in Supplementary Table S1. Statistically significant associations between radiomic variables and MRI scanning parameters are identified by a significant linear model (2), adjusted for multiple comparisons between radiomic features using false discovery rate (FDR) under significance level α = 0.05^28^. If significant in the model, LRM predictions were subtracted from the radiomic variable, providing a radiomic feature linearly unbiased from scanning protocol variables, followed by a final z-score normalization,

3 $${N}_{LRM}\left(f\right)= \left\{\begin{array}{c}{N}_{z}\left(f-\widetilde{f}\right) if \, model \, is \, significant\\ {N}_{z}\left(f\right) otherwise,\end{array}\right.$$

referred to as *LRM normalization* in the current work (Approach 2; Fig. 1b). Non-significant models are not subtracted from the data, leaving those radiomic features unadjusted except from z-score normalization. By subtracting linear dependencies of MR scanning protocol, the influence of protocol parameters on the extracted radiomic features is mitigated. Thereby, the processed radiomic features are more likely to reflect underlying biological phenotypes rather than bias from scanning protocol. Equation (2) is not only utilized for the LRM normalization by Eq. (3), but also used for measuring statistical associations between MRI scanning parameters and the individual radiomic features (Fig. 1b, right panel). Individual, MRI scanning parameters significantly predicting shape features were identified from coefficients [see Eq. (2)] for whom p < 0.05.

### Statistical methods for evaluation of performance

In the present study we also investigate the effect of different normalization methods on composition of radiomic derived clusters (i), and on prediction of DSS (ii). For (i), patients were divided into two clusters using the k-medoids clustering algorithm (kmedoids.m in MATLAB, random number generator set to ’default’). Kaplan–Meier survival plots and the log-rank test assessed differences in disease-specific survival (DSS) between patients in the different clusters. Due to lack of normality, differences in MRI scanning parameters between clusters were investigated using the non-parametric Kruskal–Wallis test (kruskalwallis.m in MATLAB) for continuous variables. Differences in categorical variables between clusters were assessed by the chi-square test (crosstab.m in MATLAB). Whenever indicated, significance level is corrected for multiple testing using FDR.

We also split the patients into three groups to assess risk profiles of the patients that change cluster between normalization methods: Groups 1 or 2 (G1 or G2) consist of patients that are consistently assigned to either cluster 1 or 2, respectively, independent of normalization method, and group 3 (G3) encompasses patients that are assigned to different clusters when changing normalization method. With this grouping, differences in DSS were pairwise assessed between the groups using the log-rank test.

Sensitivity to shrinking the number of radiomic features on clustering was tested by repeatedly applying k-medoids clustering on a dataset, each time removing a random subset of features. This was done in increments of 5% of the total features, ensuring different subsets were chosen each time by setting the random number generator to 'shuffle'. After removal of a random subset of features, the clustering algorithm was applied to the modified dataset, and the process was repeated 50 times to obtain a measure of variance. By gradually increasing the percentage of removed radiomic features, the process evaluates how the cluster groups vary as more features are eliminated. The process stops when 10% of the radiomic features remain in the data set. Choosing this method over others like principal component analysis for reducing feature dimensionality is strategic. Principal component analysis reduces dimensionality while retaining most of the variability in the data. Our approach directly addresses how the chosen number of features impacts clustering.

For investigating the effect of radiomic feature normalization on predicting DSS (ii), the study employed random survival forests (RSF) and LASSO (least absolute shrinkage and selection operator) Cox regression implemented in the scikit-survival library RandomSurvivalForest version 0.21.0^29^ In the LASSO Cox regression, a L1 ratio of 1.0 and an alpha_min_ratio of 0.01 were used. The models were trained and evaluated on 70% and 30% of the patients, respectively, using K = 20 fold cross-validation. Time-dependent area under the ROC (receiver operating characteristic) curves (tdAUC) for predicting DSS were applied to the validation data set and computed using cumulative_dynamic_auc from the scikit-survival library, providing a visual representation of the model’s performance and discriminatory power over time.

Radiomic shape features, based on the tumor mask, were included in the analyses of statistical association between MRI scanning parameters and radiomic features (Section “Association between radiomic features and MRI scanning parameters”), but excluded in the subsequent clustering (Sections “Unsupervised clustering” and “Sensitivity to shrinking the number of radiomic features in cluster analyses”), and analyses of tdAUC (Section “Prediction of disease-specific survival”). This was done due to well-known, strong associations between large tumor volume and high-risk disease^1,2^. By not including shape variables like tumor volume and other highly associated features in the prognostic modelling, we specifically focus on the less volume dependent radiomic features, making it easier to evaluate the specific contributions and validity of a broader family of radiomic features.

### Ethics approval and consent to participate

Regional Comittee for Medical Research Ethics Western Norway approved this retrospective study (#2015/2333/REK vest). All patients included in the study gave their written informed consent at the time of their primary diagnosis.

## Results

The effect of MRI protocol parameters for radiomic feature analyses was assessed in two patient cohorts with endometrial- (n = 136) and cervical (n = 132) cancer in whom MRI radiomic tumor features were compared after z-score normalization (1) or LRM normalization (3).

### Association between radiomic features and MRI scanning parameters

**Figure 2:**
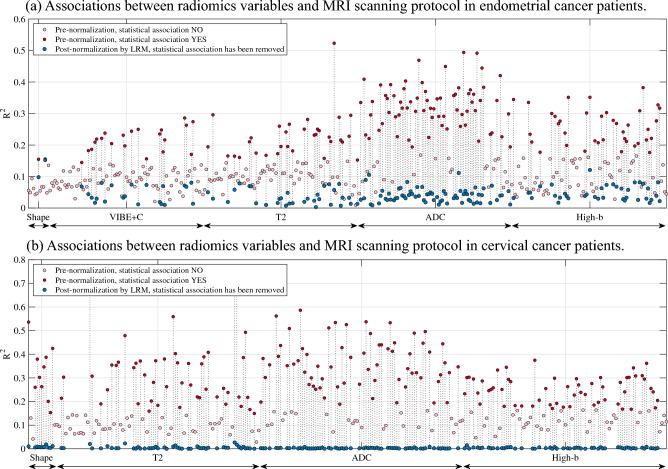
Relationship between each radiomic tumor feature (n = 385/n = 292 for EC/CC; represented as colored dots) and all MRI scanning protocol parameters (jointly) for the corresponding sequence, measured in terms of the R^2^-values of the linear regression model (2). Individual radiomic features are grouped by the MRI series (highlighted with different shades of gray along the horizontal axis) from which they are derived. Pink dots are radiomic features that show no association with MRI scanning protocol. In both cohorts, R^2^-values of the linear model remain unchanged upon z-score normalization. (**a**) In the endometrial cancer series (n = 136 patients), prior to any normalization, a substantial portion of the radiomic features was associated with MRI scanning parameters [red dots, 162/385, (42%), Eq. (2)]. (**b**) Also, in the cervical cancer series (n = 132 patients) many radiomic features have an association with MRI scanning parameters [red dots, n = 180/292 (62%), Eq. (2)]. In both cohorts, after LRM normalization, none of the radiomic features are associated with MRI scanning protocol (blue dots being connected to the red dot representing R^2^ for the same feature prior to LRM normalization). Significance level is corrected for multiple testing using false discovery rate (FDR). *ADC* apparent diffusion coefficient, *LRM* linear regression model, *T2* T2 weighed MRI, *VIBE* + *C* T1 weighted MRI with contrast.

For endometrial cancer patients, 162/385 (42%) of the radiomic features were significantly associated with scanning protocol. This is seen as multiple red dots in Fig. 2a, representing R^2^ values of the linear model in (2) demonstrating significant associations between MRI scanning parameters and individual radiomic features. These were features from (i) tumor segmentation (shape features): 2/13 (15%); (ii) VIBE + C: 21/93 (23%); (iii) T2: 32/93 (34%); (iv) ADC: 69/93 (74%); (v); and high-b: 38/93 (41%). The two significant shape features (maximum2DDiameterRow and Sphericity) were significantly predicted by voxel volume and slice thickness [Eq. (2), p < 0.05].

For cervical cancer patients, 180/292 (62%) of the radiomic features were significantly associated with scanning protocol (Fig. 2b, red dots). These were features from (i) tumor segmentation (shape features): 10/13 (77%); (ii) T2: 48/93 (52%); (iii) ADC: 66/93 (71%); and (iv) high-b: 56/93 (60%). The 10 significant shape features were predicted by [Eq. (2), p < 0.05]: voxel volume (8 out of 10), slice thickness (7 out of 10), anisotropy (8 out of 10), field of view (9 out of 10), and phase-encoding direction (3 out of 10).

The dependencies between MRI protocol and radiomic features are unaffected by z-score normalization, leaving all R^2^-values unchanged after z-score normalization. After LRM normalization, none of the radiomic features were associated with MRI scanning parameters (Fig. 2, blue dots).

### Unsupervised clustering

**Figure 3:**
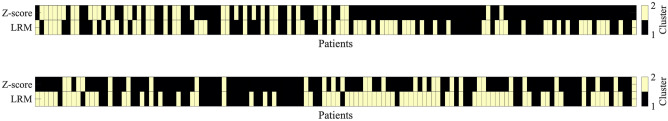
Unsupervised clustering of radiomic features after normalization by z-score and LRM shows that choice of normalization method largely impacts the clustering. Top: In the endometrial cancer cohort (n = 136), 49/136 (36%) of the patients changed cluster between normalization methods. Bottom: In the cervical cancer cohort (n = 132), 50/132 (38%) of the patients changed cluster between normalization methods. Each patient is represented by a vertical bar, and the colour of the bar indicates cluster assignment. *LRM* linear regression model.

Based on radiomic features, endometrial cancer patients (n = 136) were clustered into two radiomic clusters, either after z-score normalization [Cluster 1: 97/136 (71%); Cluster 2: 39/136 (29%)], or after LRM normalization [Cluster 1: 68/136 (50%); Cluster 2: 68/136 (50%)]. Altogether, 49/136 (36%) of the endometrial cancer patients changed cluster between normalization method (Fig. 3a). Supplementary Table S2 presents MRI scanning parameter dependencies within the two radiomic endometrial cancer clusters after z-score- and LRM normalization of the radiomic variables, respectively. After z-score normalization, 27/31 (87%) of the MRI scanning parameters were over-represented in one of the clusters, whereas after LRM normalization, none of the MRI parameters were over-represented in any of the clusters.

Radiomic clustering of cervical cancer patients (n = 132) yielded two radiomic cluster groups after z-score normalization [Cluster 1: 101/132 (77%); Cluster 2: 31/132 (24%)], and after LRM normalization [Cluster 1: 55/132 (42%); Cluster 2: 77/132 (58%)]. Altogether, 50/132 (38%) of the cervical cancer patients changed cluster between normalization method (Fig. 3b). After z-score normalization, 7/22 (32%) of the MRI scanning parameters were over-represented in any of the two clusters, whereas after LRM normalization, none of the MRI scanning parameters were over-represented in any of the clusters (Supplementary Table S3).

**Figure 4:**
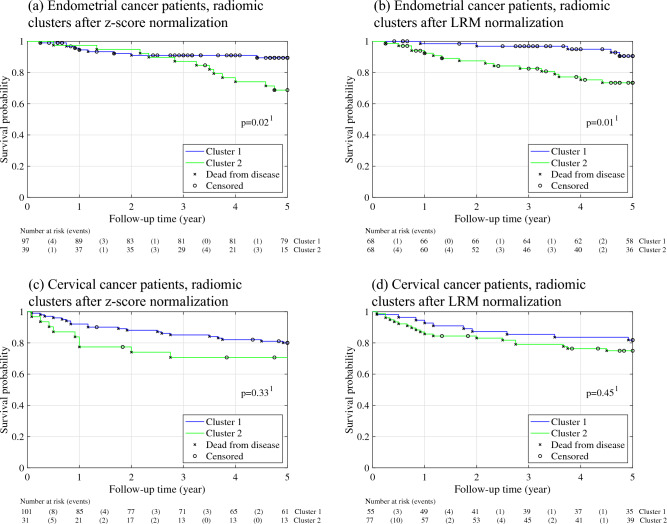
Kaplan–Meier plots depicting disease-specific survival for patients belonging to the two radiomic clusters. (**a**, **b**): Endometrial cancer patients. Survival probability for clusters after (**a**) Z-score normalized data (log-rank test: p = 0.02) and (**b**) LRM normalized data (log-rank test: p = 0.01). Both normalization methods identify two distinct patient groups exhibiting significantly different survival. (**c**, **d**): Cervical cancer patients. Survival probability of (**c**) Z-score normalized data (log-rank test: p = 0.33) and (**d**) LRM normalized data set (log-rank test: p = 0.45). After normalization by both methods, the derived patient clusters exhibit similar survival. *LRM* linear regression model. ^1^log-rank test for differences in disease specific survival between clusters.

In endometrial cancer patients, the two patient clusters generated after z-score- and LRM normalization, respectively, exhibited similarly different DSS for patients in cluster 1 vs 2 (log-rank test; Fig. 4a, z-score: p = 0.02; Fig. 4b, LRM: p = 0.01). Also in the cervical cancer patients, the radiomic patient clusters exhibited similar DSS irrespective of normalization method (log-rank test; Fig. 4c, z-score: p = 0.33; Fig. 4d, LRM: p = 0.45).

Endometrial cancer patients always belonging to Cluster 1 (G1), irrespective of normalization method, had better DSS than patients always belonging to Cluster 2 (G2) (log-rank test; G1 vs. G2: p = 0.002) (Fig. S1, left). Patients changing radiomic cluster (G3) between normalization methods had similar DSS to that of G1 and G2 (Fig. S1, left). No difference in DSS was seen for cervical cancer patients between the three groups G1, G2, and G3 (Fig. S1, right).

### Sensitivity to shrinking the number of radiomic features in cluster analyses

**Figure 5:**
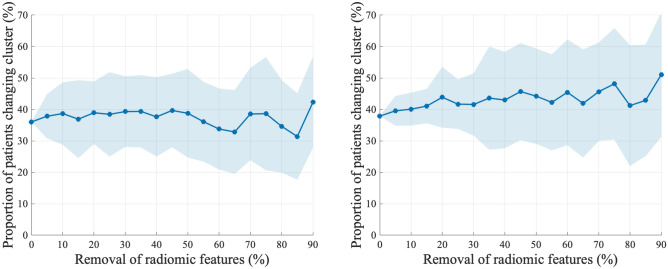
Proportion (%) of patients changing clusters between the two normalization methods. Prior to clustering, radiomic features are randomly removed stepwise by [0%, 5%, …, 90%] of the total radiomic features. Clustering is repeated n = 50 times for each step, providing a standard deviation (SD) of the estimate (shaded area). Left: Endometrial cancer (n = 136 patients). The plot suggests that a high proportion (mean between 31 and 43%) of the patients changes cluster between the two normalization methods, irrespective of the percentage of radiomic features (between 10 and 100%) included in the model. Right: Cervical cancer (n = 132 patients). A high proportion (mean between 38 and 51%) of the patients changes cluster between the two normalization methods. As radiomic features are removed, the variation of patients changing clusters tends to increase. In both data sets, normalization method largely impacts clustering composition.

Figure 5 depicts the proportion (and standard deviation) of patients changing cluster between the two normalization methods, when the cluster analyses are performed with a decreasing number of (randomly selected) radiomic features (shrinking features from 0 to 90%). For endometrial cancer patients, the average proportion of patients changing cluster between normalization methods, is mostly stable and varies between 31 and 43% when removing 0–90% of radiomic features (Fig. 4, left). For cervical cancer patients, the average proportion of patients changing cluster between normalization method is slightly higher but also stable, however with increasing standard deviation, and varies from 38 to 51% (Fig. 4, right).

### Prediction of disease-specific survival

**Figure 6:**
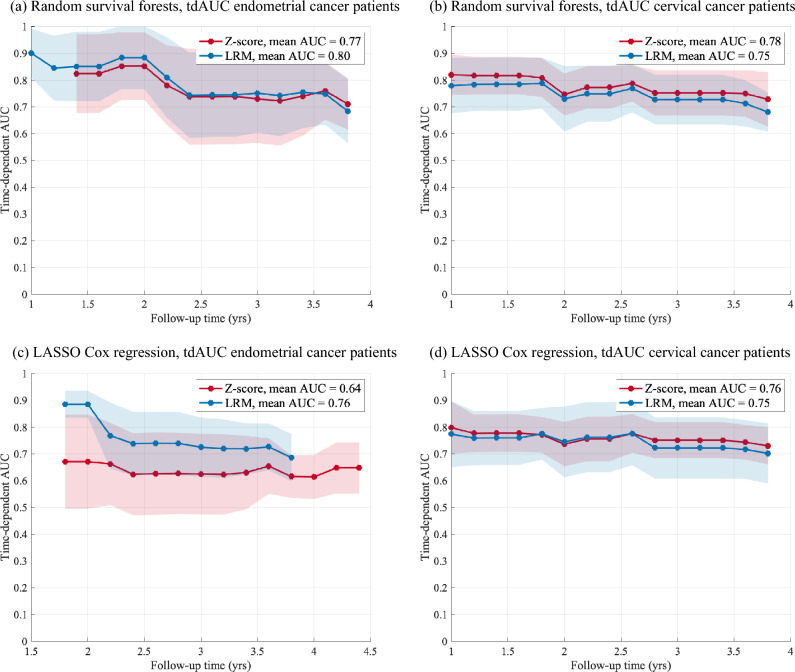
Time-dependent area under the ROC curve (tdAUC) for predicting disease specific survival (DSS) in validation data sets (n = 41 endometrial cancer patients, n = 40 cervical cancer patients) based on tumor radiomic features using z-score (red) and LRM (blue) normalization. (**a**) Endometrial cancer patients, random survival forests, (**b**) Cervical cancer patients, LASSO Cox regression, (**c**) Endometrical cancer patients, LASSO Cox regression, (**d**) Cervical cancer patients, LASSO Cox regression. In both patient groups, the two normalization methods yield similar tdAUC for predicting DSS based on radiomic tumor profiles using random survival forests. However, when using LASSO Cox regression for predicting DSS in endometrial cancer patients, the performance was somewhat higher for LRM normalized data than for Z-score normalized data (mean ADC of 0.76 compared to 0.64). This difference in performance was not visible for cervical cancer patients. Shaded area is the standard deviation across K = 20 folds. *LASSO* least absolute shrinkage and selection operator, *LRM* linear regression model, *ROC* receiver operating characteristics.

Using random survival forests and LASSO Cox regression, tdAUCs for predicting DSS based on radiomic features after z-score/LRM normalization are presented in Fig. 6. For both cancers, there is no significant difference in mean tdAUCs for predicting DSS over time using random survival forests (endometrial cancer: mean tdAUC = 0.77/0.80 (Fig. 6a); cervical cancer: mean tdAUC = 0.78/0.75 (Fig. 6b) by z-score-/LRM normalized radiomic features). Using LASSO Cox regression, z-score/LRM normalized radiomic features yielded mean tdAUCs of 0.64/0.76 and 0.76/0.75 in endometrial (Fig. 6c) and cervical cancer (Fig. 6d), respectively, for predicting DSS.

## Discussion

For radiomic tumor profiling to become clinically useful and comparable across centres and patients, the profiles should ideally capture biologically relevant tumor features potentially reflected in clinical phenotypes. The current study shows that a large proportion (42%/62%) of the radiomic features in the two endometrial/cervical cancer cohorts is statistically associated with MRI scanning parameters. Furthermore, the observed dependencies between MRI scanning parameters and radiomic feature values are not eliminated by the widely used z-score normalization method. However, when utilizing a normalization approach that incorporates MRI scanning parameters in a linear regression model (LRM), all extracted radiomic variables become unbiased by MRI scanning parameters. Thus, this study shows that LRM normalization, as opposed to z-score normalization, effectively eliminates the linear relationship between radiomic tumor features and MRI scanning parameters.

ComBat normalization, initially developed for genomics, was adapted for multicenter radiomics studies to mitigate batch differences using a negative binomial regression model^23^. Using ComBat normalization of tumor radiomic features has shown to improve their predictive and prognostic power in cervical cancer. Interestingly, our linear regression model extends the approach used in ComBat employing batchwise normalization, to also incorporate continuous variables, thus offering a more versatile solution for normalizing radiomics data across scanners and imaging protocols.

In order to utilize multiparametric MRI-derived radiomics for prediction of clinical phenotype, different statistical approaches may be used. In the present study we investigated the effect of normalization method on composition of derived radiomic clusters (applying a k-medoids clustering algorithm), and on prediction of survival based on radiomic profiles (using random survival forests and LASSO Cox regression). The composition of radiomic clusters was to a high degree affected by choice of normalization method (z-score vs. LRM) in both of the investigated cohorts, and a large proportion of patients changed clusters (31%-51%), between normalization methods. In addition, many of MRI scanning parameters were unevenly distributed across the two clusters following z-score normalization, implying that MRI scanning parameters significantly impact the extracted radiomic feature values and the subsequent radiomic clustering. Interestingly, although the cluster composition was highly affected by the choice of normalization method, the observed difference in DSS for the two radiomic clusters in endometrial cancer was relatively similar. A possible explanation may be that if radiomic tumor features do reflect clinical phenotypes, they are captured despite MRI scanning protocol bias. However, the demonstrated lack of robustness for cluster allocation, clearly limits its potential use to guide individualized treatment schemes.

The optimal number of radiomic features for modelling is unclear. Shrinking the number of features in the endometrial cancer cohort did not significantly change patient clustering, indicating feature redundancy. This supports the use of fewer, reproducible features to avoid over-fitting. However, in the cervical cancer cohort, reducing features caused higher clustering variability between normalization methods. Even in this scenario prone to over-fitting, when number of radiomic features exceed the number of patients^30^, reducing the radiomic features decreased clustering reproducibility in cervical cancer. This difference in stability of patient clustering when shrinking the number of features in endometrial and cervical cancers can be due the more inhomogeneous MRI scanning protocols used in the cervical cancer cohort.

In general, the predictive modelling by random survival forests and LASSO Cox regression both performed relatively well with moderate to high tdAUC for predicting DSS. Using random survival forests yielded tdAUCs of 0.75–0.80 for predicting DSS in cervical and endometrial cancers, suggesting that random survival forests are quite robust, irrespective of normalization method. In comparison, the LASSO Cox regression model yielded slightly lower tdAUC for Z-score normalized features (tdAUC: 0.64) than for LRM normalized features (tdAUC: 0.76) in endometrial cancer, whereas similar tdAUCs (of 0.76/0.75 for Z-score/LRM normalized radiomic features) in cervical cancer, suggesting that the LASSO Cox regression model may be more sensitivity to differences in normalization method. The apparent discrepancy in performance between the two models is likely to be attributable to inherent architectural differences between the models. Whereas the random survival forest model is a non-linear machine learning approach effectively capturing non-linear patterns within the data, the LASSO Cox regression operates on a linear premise with respect to the data, potentially limiting its adaptability to complex and multifaceted radiomic data in the prediction of DSS.

Unlike unsupervised clustering methods, random survival forests and LASSO Cox regression are supervised models trained on clinical outcomes. Hence, inherent biases in the data material, that do not improve prognostication, may be treated as noise by the random survival forests. This might also explain an apparent resilience to biases from scanning parameters when using the random survival forest model, as opposed to unsupervised clustering.

Radiomic shape features, generated based on manually segmented tumor mask, would expectedly be only minimally biased by MRI scanning settings. Nevertheless, a surprisingly high proportion of the shape features [15% (2/13) in the endometrial and 77% (10/13) in the cervical cancers], was statistically associated with MRI scanning protocol in the present study. One possible explanation could be that MRI scanning parameters indirectly influence the radiologists’ ability to accurately delineate the tumor borders. Drawing tumor masks on contrast-enhanced VIBE images in endometrial cancer may also have been easier than on T2-weighted images in cervical cancer due to the very high conspicuity of tumor borders on VIBE images. It is also possible that a coarse vs. fine discretization of the tumor mask affects the computation of shape features differently. Finally, field of view is associated with many shape features, which may be due to large tumors requiring extended field of view in order to visualize the entire tumor. Based on the present findings, it is essential to recognize and address potential biases also in relation to the manual tumor segmentation, when designing future radiomic studies.

We acknowledge that the current study has several limitations. Deliberately, we tested and compared models being unsupervised (clustering with subsequent log-rank test) and supervised (random survival forests and LASSO Cox regression), in order to describe the variability and robustness when using different prediction models. The use of other models could have identified other differences between the normalization methods. We also restricted the study to two common gynecological cancers, endometrial- and cervical cancers, with MRI acquisitions of the pelvis only. Biases in MRI acquisitions of extrapelvic body organs or tumors may behave differently. Finally, possible non-perfect alignment between the DWI series and the series used for tumor segmentations, may have led to slightly incorrect placement of tumor mask on the DWI, which may have influenced the DWI radiomic features.

In summary, this study demonstrates substantial MRI scanning parameter bias on z-score normalized radiomic features, whereas no bias after LRM normalization, in two well-annotated gynecologic cancer patient cohorts. Importantly, bias from MRI scanning parameters impacts radiomic clustering and cluster compositions. However, z-score- and LRM normalized tumor radiomic features seem to yield equal prognostic power for predicting DSS in endometrial- and cervical cancer patients, suggesting that the algorithms utilized are robust enough to capture prognostic radiomic profiles, despite MRI scanning parameter bias. Importantly, standardization of MRI protocols and adequate normalization of radiomic features are clearly needed if radiomic tumor profiling is to be introduced for prognostication and treatment tailoring in cancers.

### Supplementary Information

Supplementary Legends.

Supplementary Figure S1.

Supplementary Table S1.

Supplementary Table S2.

Supplementary Table S3.

## Data Availability

Due to privacy regulations outlined in the study's approval, the primary data supporting the findings of this research are not publicly accessible. However, secondary data could be made available by the corresponding author upon reasonable request and subject to the data owner's approval.
