# Supplementary material for: Impact of MRI radiomic feature normalization for prognostic modelling in uterine endometrial and cervical cancers
Source: Sci Rep. 2024 Jul 22;14:16826. doi: 10.1038/s41598-024-66659-w (PMC11263557; doi:10.1038/s41598-024-66659-w)
Supplement: Supplementary file 1 — Supplementary Legends. [file 41598_2024_66659_MOESM1_ESM.docx]

SUPPLEMENTARY MATERIAL SECTION

LEGENDS

Figure S1 Kaplan-Meier plots depicting DSS for patients always belonging to cluster 1 G1), cluster 2 (G2), or those that change cluster based on normalization method (G3). Left: Endometrial cancer. Patients always in cluster 1 have better prognosis than patients always in cluster 2. Patients changing cluster have similar DSS to those always in cluster 1/2 (log-rank test; G1 vs. G2: p=0.002; G1 vs. G3: p=0.30; G2 vs. G3: p=0.10). Right: Cervical cancer. There is no statistical diﬀerence in DSS between any of the groups (log-rank test; G1 vs. G2: p=0.48; G1 vs. G3: p=0.94; G2 vs. G3: p=0.61). Although not statistically signiﬁcant, in both cohorts, the patient group who changed cluster tends to associate with intermediate DSS. DSS=disease speciﬁc survival; vs.=versus.
