# Supplementary figures and images for: Impact of MRI radiomic feature normalization for prognostic modelling in uterine endometrial and cervical cancers
Source: Sci Rep. 2024 Jul 22;14:16826. doi: 10.1038/s41598-024-66659-w (PMC11263557; doi:10.1038/s41598-024-66659-w)

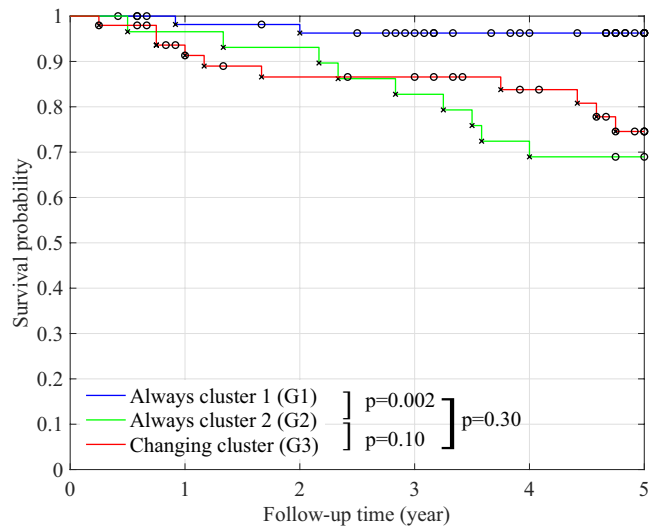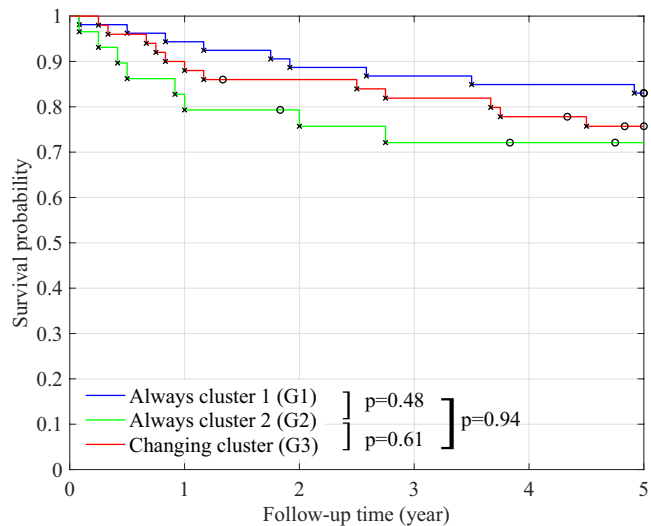

Supplement: Supplementary file 2 — Supplementary Figure S1. [file 41598_2024_66659_MOESM2_ESM.pdf]
