# Supplementary material for: Impact of MRI radiomic feature normalization for prognostic modelling in uterine endometrial and cervical cancers
Source: Sci Rep. 2024 Jul 22;14:16826. doi: 10.1038/s41598-024-66659-w (PMC11263557; doi:10.1038/s41598-024-66659-w)
Supplement: Supplementary file 3 — Supplementary Table S1. [file 41598_2024_66659_MOESM3_ESM.docx]

Table S1 Pelvic MRI scanning protocol parameters for endometrial (n=136) - and cervical (n=132) cancer patients undergoing MRI radiomic tumor proﬁling. Protocol settings are categorized and counted (percentage out of total patient numbers in brackets) within deﬁned intervals and are indicated as being either primary- (in bold) or secondary (in italic, derived from primary variables) MRI scanning protocol variables. Empty cells indicate non-existing variable for a given MRI series.

**Endometrial cancer (n=136) Cervical cancer (n=132)**

| MRI scanning parameter | Interval^4^ | VIBE+C (%) | T2 (%) | DWI (%) | T2 (%) | DWI (%) |
| --- | --- | --- | --- | --- | --- | --- |
| **Spacing** [mm] | [0.3, 1.1) | 65 (48) | 136 (100) | 0.0 | 132 (100) | 10 (8) |
|  | [1.1, 2) | 71 (52) | 0.0 | 14 (10) | 0.0 | 92 (70) |
|  | [2, 2.8] | 0.0 | 0.0 | 122 (90) | 0.0 | 30 (23) |
| **Matrix** | [100, 243) | 80 (59) |  | 136 (100) |  | 91 (69) |
|  | [243, 385) | 56 (41) | 136 (100) |  | 55 (42) | 41 (31) |
|  | [385, 528] |  |  |  | 77 (58) |  |
| **TR**^1^ [ms] | [0, 2770) | 136 (100) |  |  | 9 (7) | 24 (18) |
|  | [2770, 5540) |  | 69 (51) | 78 (57) | 115 (87) | 89 (67) |
|  | [5540, 8310] |  | 67 (49) | 58 (43) | 8 (6) | 19 (14) |
| **TE**^1^ [ms] | [2, 45) | 136 (100) |  |  |  |  |
|  | [45, 87) |  |  | 131 (96) | 13 (10) | 132 (100) |
|  | [87, 130] |  | 136 (100) | 5 (4) | 119 (90) |  |
| **FA**^1^ [◦] | [0, 135) | 136 (100) | 1 (1) | 71 (52) | 41 (31) | 108 (82) |
|  | [135, 180] |  | 135 (99) | 65 (48) | 91 (69) | 24 (18) |
| **Field strength**^1,3^ [T] | 1.5 | 71 (52) | 71 (52) | 71 (52) | 95 (72) | 95 (72) |
|  | 3 | 65 (48) | 65 (48) | 65 (48) | 37 (28) | 37 (28) |
| **Slice thickness**^1^ [mm] | [1, 2) | 65 (48) |  |  |  |  |
|  | [2, 4) | 71 (52) | 136 (100) | 65 (48) | 115 (87) | 50 (38) |
|  | [4, 8] |  |  | 71 (52) | 17 (13) | 82 (62) |
| **Number of averages**^1,2^ | [1, 4) | 136 (100) | 136 (100) | 65 (48) | 104 (79) | 63 (48) |
|  | [4, 12] |  |  | 71 (52) | 28 (21) | 69 (52) |
| **Number of slices** | [14, 44) |  | 136 (100) | 136 (100) | 132 (100) | 132 (100) |
|  | [44, 74) | 71 (52) |  |  |  |  |
|  | [74, 104] | 65 (48) |  |  |  |  |
| **Phase-encoding direction**^1,3^ | ROW | 80 (59) | 81 (60) | 66 (49) | 113 (86) | 41 (31) |
|  | COLUMN | 56 (41) | 55 (40) | 70 (51) | 19 (14) | 91 (69) |
| *Voxel volume*^1^ [mm^3^] | [0, 13) | 136 (100) | 136 (100) | 64 (47) | 132 (100) | 101 (77) |
|  | [13, 25) |  |  | 1 (1) |  | 16 (12) |
|  | [25, 38] |  |  | 71 (52) |  | 15 (11) |
| *Anisotropy*^1^ | [0, 2) | 136 (100) | 0.0 | 51 (38) | 0.0 | 43 (33) |
|  | [2, 5) | 0.0 | 71 (52) | 85 (62) | 15 (11) | 77 (58) |
|  | [5, 7] | 0.0 | 65 (48) | 0.0 | 39 (30) | 12 (9) |
| *FOV*^1^ [cm^2^] | [250, 500) |  | 135 (99) | 64 (47) | 131 (99) | 30 (23) |
|  | [500, 1690] | 136 (100) | 1 (1) | 72 (53) | 1 (1) | 102 (77) |
| **Low-b** [s·mm^-2^] | [0, 25) |  |  | 128 (94) |  | 79 (60) |
|  | [25, 75) |  |  |  |  | 53 (40) |
|  | [75, 500] |  |  | 8 (6) |  |  |
| **High-b**^1^ [s·mm^-2^] | [800, 1000) |  |  |  |  | 36 (27) |
|  | [1000, 4000] |  |  | 136 (100) |  | 96 (73) |
| **Number of b-values**^1^ | [2, 3) |  |  | 86 (63) |  | 38 (29) |
|  | [3, 19] |  |  | 50 (37) |  | 94 (71) |

DWI=diﬀusion weighted imaging; FA=ﬂip angle; FOV=ﬁeld of view; T=Tesla; TE=echo time; TR=repetition time; T2=T2-weighted MRI; VIBE+C=T1-weighted imaging with contrast.

^1^Used in the linear regression model for normalization. High b-value and number of b-values were only used in the linear regression

model of DWI derived variables.

^2^Not used in the linear regression model for normalization for VIBE+C derived radiomic variables.

^3^Categorical variables.

^4^Half-open intervals (left or right) include only one of the bounds.
