# Supplementary material for: Impact of MRI radiomic feature normalization for prognostic modelling in uterine endometrial and cervical cancers
Source: Sci Rep. 2024 Jul 22;14:16826. doi: 10.1038/s41598-024-66659-w (PMC11263557; doi:10.1038/s41598-024-66659-w)
Supplement: Supplementary file 4 — Supplementary Table S2. [file 41598_2024_66659_MOESM4_ESM.docx]

Table S2 Table values report median of MRI scanning protocol parameters within clusters C1 and C2 (counts (%) for categorical variables∗) for endometrial cancer patients. Signiﬁcant diﬀerences between clusters are indicated with bold p-values, corrected for multiple testing using FDR (false discovery rate). Physical units are provided in brackets, whenever present.

**Z-score LRM**

| Modality | MRI scanning parameter | C1 (n=97) | C2 (n=39) | p | | C1 (n=68) | | C2 (n=68) | p |
| --- | --- | --- | --- | --- | --- | --- | --- | --- | --- |
| VIBE | Voxel volume [mm^3^] | 1.14 | 3.39 | **<0.001**^1^ | | 3.39 | | 3.39 | 0.973^1^ |
|  | Anisotropy | 1.23 | 1.54 | | **<0.001**^1^ | 1.54 | | 1.54 | 0.643^1^ |
|  | TR [ms] | 5.86 | 7.23 | | **<0.001**^1^ | 7.23 | | 7.23 | 0.806^1^ |
|  | TE [ms] | 2.46 | 2.55 | | **<0.001**^1^ | 2.55 | | 2.55 | 0.864^1^ |
|  | FA [◦] | 9 | 16.4 | | **<0.001**^1^ | 14.5 | | 14.6 | 0.790^1^ |
|  | Field of view [cm^2^] | 566 | 625 | | **0.001**^1^ | 625 | | 625 | 0.211^1^ |
|  | Slice thickness [mm] | 1.2 | 2 | | **<0.001**^1^ | 2 | | 2 | 0.510^1^ |
|  | Field strength-1.5 [T]* | 34 (35) | 37 (95) | | **<0.001**^2^ | 35 (51) | | 36 (53) | 0.864^2^ |
|  | Field strength-3 [T]* | 63 (65) | 2 (5) | |  | 33 (49) | | 32 (47) |  |
|  | Phase-encoding direction-COLUMN* | 34 (35) | 22 (56) | | **0.022**^2^ | 24 (35) | | 32 (47) | 0.163^2^ |
|  | Phase-encoding direction-ROW* | 63 (65) | 17 (44) | |  | 44 (65) | | 36 (53) |  |
| T2 | Voxel volume [mm^3^] | 0.814 | 1.48 | **<0.001**^1^ | | 1.48 | | 1.48 | 0.923^1^ |
|  | Anisotropy | 5.76 | 4.27 | | **<0.001**^1^ | 4.27 | | 4.27 | 0.801^1^ |
|  | TR [s] | 4.44 | 6.31 | | **<0.001**^1^ | 5.56 | | 4.91 | 0.297^1^ |
|  | TE [ms] | 94 | 95 | | **<0.001**^1^ | 95 | | 95 | 0.864^1^ |
|  | FA [◦] | 150 | 150 | | 0.269^1^ | 150 | | 150 | 0.561^1^ |
|  | Field of view [cm^2^] | 400 | 324 | | **<0.001**^1^ | 324 | | 324 | 0.869^1^ |
|  | Slice thickness [mm] | 3 | 3 | | 0.526^1^ | 3 | | 3 | 0.317^1^ |
|  | Field strength-1.5 [T]* | 34 (35) | 37 (95) | | **<0.001**^2^ | 35 (51) | | 36 (53) | 0.864^2^ |
|  | Field strength-3 [T]* | 63 (65) | 2 (5) | |  | 33 (49) | | 32 (47) |  |
|  | Phase-encoding direction-COLUMN* | 34 (35) | 21 (54) | | **0.043**^2^ | 26 (38) | | 29 (43) | 0.600^2^ |
|  | Phase-encoding direction-ROW* | 63 (65) | 18 (46) | |  | 42 (62) | | 39 (57) |  |
|  | Number of averages | 2 | 2 | | 0.611^1^ | 2 | | 2 | 0.368^1^ |
| DWI | Voxel volume [mm^3^] | 12 | 27.5 | **<0.001**^1^ | | 27.5 | 27.5 | | 0.761^1^ |
|  | Anisotropy | 1.6 | 2.13 | | **0.002**^1^ | 2.13 | | 2.13 | 0.636^1^ |
|  | TR [s] | 5.56 | 3.1 | | **<0.001**^1^ | 3.1 | | 3.1 | 0.632^1^ |
|  | TE [ms] | 77 | 79 | | **<0.001**^1^ | 79 | | 79 | 0.676^1^ |
|  | FA [◦] | 180 | 90 | | **<0.001**^1^ | 90 | | 90 | 0.864^1^ |
|  | Field of view [cm^2^] | 400 | 900 | | **<0.001**^1^ | 900 | | 900 | 0.800^1^ |
|  | Slice thickness [mm] | 3 | 5 | | **<0.001**^1^ | 5 | | 5 | 0.928^1^ |
|  | Field strength-1.5 [T]* | 34 (35) | 37 (95) | | **<0.001**^1^ | 35 (51) | | 36 (53) | 0.864^2^ |
|  | Field strength-3 [T]* | 63 (65) | 2 (5) | |  | 33 (49) | | 32 (47) |  |
|  | Phase-encoding direction-COLUMN* | 46 (47) | 24 (62) | | 0.136^2^ | 29 (43) | | 41 (60) | 0.040^2^ |
|  | Phase-encoding direction-ROW* | 51 (53) | 15 (38) | |  | 39 (57) | | 27 (40) |  |
|  | Number of averages | 1 | 12 | | **<0.001**^1^ | 12 | | 12 | 0.977^1^ |
|  | High-b [s·mm^-2^] | 1000 | 1000 | | **0.038**^1^ | 1000 | | 1000 | 0.488^1^ |
|  | Number of b-values | 2 | 2 | | **<0.001**^1^ | 2 | | 2 | 0.750^1^ |

DWI=diffusion weighted imaging; FA=flip angle; LRM=linear regression model; T=Tesla; TE=echo time; TR=repetition time; T2=T2-weighted MRI; VIBE+C=T1-weighted imaging with contrast.

^1^Kruskal-Wallis non-parametric analysis of variance (ANOVA)

^2^Chi-square statistical test
