# Supplementary material for: Impact of MRI radiomic feature normalization for prognostic modelling in uterine endometrial and cervical cancers
Source: Sci Rep. 2024 Jul 22;14:16826. doi: 10.1038/s41598-024-66659-w (PMC11263557; doi:10.1038/s41598-024-66659-w)
Supplement: Supplementary file 5 — Supplementary Table S3. [file 41598_2024_66659_MOESM5_ESM.docx]

Table S3 Table values report median of MRI scanning protocol parameters within clusters C1 and C2 (counts (%) for categorical variables∗) for cervical cancer patients. Signiﬁcant diﬀerences between clusters are indicated with bold p-values, corrected for multiple testing using FDR (false discovery rate). Physical units are provided in brackets, whenever present.

**Z-score LRM**

| Modality | MRI scanning parameter | | C1 (n=56) | C2 (n=76) | p | C1 (n=110) | C2 (n=22) | p |
| --- | --- | --- | --- | --- | --- | --- | --- | --- |
| T2 | Voxel volume [mm^3^] | | 0.481 | 0.458 | 0.430 | 0.458 | 0.763 | 0.048^1^ |
|  | Anisotropy | | 7.49 | 7.11 | 0.948^1^ | 8.53 | 7.11 | 0.079^1^ |
|  | TR [s] | | 4.61 | 4.03 | **<0.001**^1^ | 4.45 | 4.61 | 0.561^1^ |
|  | TE [ms] | | 100 | 95 | 0.042^1^ | 100 | 98 | 0.455^1^ |
|  | FA [◦ ] | | 150 | 150 | 0.255^1^ | 150 | 150 | 0.876^1^ |
|  | Field of view [cm2] | | 324 | 324 | 0.129^1^ | 324 | 400 | 0.263^1^ |
|  | Slice thickness [mm] | | 3 | 3 | 0.262^1^ | 3 | 3 | 0.414^1^ |
|  | Field strength-1.5 [T]* | | 76 (75) | 19 (61) | 0.130^2^ | 43 (78) | 52 (68) | 0.179^2^ |
|  | Field strength-3 [T]* | | 25 (25) | 12 (39) |  | 12 (22) | 25 (32) |  |
|  | Phase-encoding direction-COL* | | 13 (13) | 6 (19) | 0.368^2^ | 10 (18) | 9 (12) | 0.295^2^ |
|  | Phase-encoding direction-ROW* | | 88 (87) | 25 (81) |  | 45 (82) | 68 (88) |  |
|  | Number of averages | | 2 | 2 | 0.055^1^ | 2 | 2 | 0.421^1^ |
| DWI | Voxel volume [mm3] |  | 10.1 | 8.07 | 0.412^1^ | 7.32 | 10.7 | 0.187^1^ |
|  | Anisotropy | | 2.16 | 3.03 | **0.002**^1^ | 2.16 | 2.4 | 0.887^1^ |
|  | TR [s] | | 3.20 | 4.02 | **<0.001**^1^ | 3.22 | 3.28 | 0.225^1^ |
|  | TE [ms] | | 79 | 68.7 | **0.011**^1^ | 79 | 74 | 0.029^1^ |
|  | FA [◦ ] | | 90 | 90 | 0.847^1^ | 90 | 90 | 0.171^1^ |
|  | Field of view [cm2] | | 562 | 900 | 0.151^1^ | 562 | 784 | 0.863^1^ |
|  | Slice thickness [mm] | | 4 | 4 | 0.048^1^ | 4 | 4 | 0.302^1^ |
|  | Field strength-1.5 [T]* | | 76 (75) | 19 (61) | 0.130^2^ | 43 (78) | 52 (68) | 0.179^2^ |
|  | Field strength-3 [T]* | | 25 (25) | 12 (39) |  | 12 (22) | 25 (32) |  |
|  | Phase-encoding direction-COL* | | 66 (65) | 25 (81) | 0.107^2^ | 35 (64) | 56 (73) | 0.266^2^ |
|  | Phase-encoding direction-ROW* | | 35 (35) | 6 (19) |  | 20 (36) | 21 (27) |  |
|  | Number of averages | | 6 | 2 | **0.005**^2^ | 6 | 3 | 0.253^2^ |
|  | High-b [s·mm2] | | 1000 | 1000 | **0.003**^1^ | 1000 | 1000 | 0.018^1^ |
|  | Number of b-values | | 3 | 4 | **0.002**^1^ | 3 | 3 | 0.884^1^ |

DWI=diffusion weighted imaging; FA=flip angle; LRM=linear regression model; T=Tesla; TE=echo time; TR=repetition time; T2=T2-weighted MRI; VIBE+C=T1-weighted imaging with contrast.

^1^Kruskal-Wallis non-parametric analysis of variance (ANOVA)

^2^Chi-square statistical test
